# Stakeholder perspectives on telemedicine acceptance and experiences in Uganda: A qualitative study

**DOI:** 10.1371/journal.pgph.0007047

**Published:** 2026-10-07

**Authors:** Irene Semakula, Sande Slivesteri, Benjamin Mwesige, Mukooza Edward, Benedict Akimana, Vincent Micheal Kiberu

**Affiliations:** 1 Uganda National Council for Science and Technology (UNCST), Kampala, Uganda; 2 Uganda Christian University, Mukono, Uganda; 3 Makerere University Joint AIDS Program (MJAP), Kampala, Uganda; 4 Makerere University School of Public Health, Kampala, Uganda; 5 Uganda Cancer Institute, Kampala, Uganda; 6 Department of Epidemiology and Biostatistics, Makerere University School of Public Health, Kampala, Uganda; 7 Butabika National Referral Mental Hospital, Butabika, Wakiso, Uganda; University of Embu, KENYA

## Abstract

In Uganda and other low- and middle-income countries, access to healthcare is constrained by shortages of health workers, long travel distances, and inadequate infrastructure. Telemedicine offers a potential solution, but its effectiveness depends on how key stakeholders perceive, accept, and experience it. This study explored the acceptance and experiences of telemedicine by policymakers, healthcare providers, and service users in Kampala, Uganda. An exploratory qualitative study was conducted between November 2024 and February 2025 at four sites in Kampala: Rocket Health, Uganda Cancer Institute, Butabika National Referral Mental Hospital, and the Ministry of Health. Participants were purposively selected to ensure direct experience with telemedicine. Data was collected through 15 key informant interviews with policymakers and healthcare providers and three focus group discussions with service users (patients and caregivers) who had accessed telemedicine within the previous year. Interviews and discussions were conducted in English or Luganda, audio-recorded with consent, and transcribed verbatim. Data were analyzed thematically using Braun and Clarke’s six-phase approach, supported by NVivo 12 software. Three main themes emerged: perceived benefits, perceived challenges, and recommendations for improving telemedicine. Participants described telemedicine as convenient, reducing travel and contributing to cost and time savings. It also enhanced continuity of care, flexibility, privacy, and autonomy. These benefits were particularly evident among people with disabilities and digitally literate urban users. However, challenges included poor internet connectivity, limited infrastructure, low digital literacy, concerns about diagnostic accuracy without physical examination, and variable levels of trust, with some participants preferring in-person interaction. Telemedicine was perceived as acceptable and useful. However, its broader adoption is limited by structural, technical, and trust-related barriers. Addressing these challenges will require investment in infrastructure, strengthened digital literacy, provider training, increased public awareness, and a robust national telemedicine policy to support equitable and sustainable integration into healthcare delivery in Uganda.

## Introduction

In many Low- and Middle-Income Countries (LMICs), healthcare systems face persistent challenges in providing timely and equitable care [1]. In Uganda, these challenges are driven by limited infrastructure, shortages of qualified health workers, high healthcare costs, and significant geographical barriers, particularly in rural and underserved areas where most of the population resides [2]. As a result, patients often experience long travel times, high indirect treatment costs, and unequal access to specialized services [3,4]

Uganda’s healthcare system is organized as a tiered referral structure, ranging from Village Health Teams (VHTs) at community level (Health Centre I) through Health Centres II, III and IV, to general Hospitals, regional referral hospitals, and national referral hospitals [5,6]. Recently, Community Health Extension Workers (CHEWs) have been introduced and rolled out to strengthen service delivery at the community level by supporting and supervising VHTs [7,8]. Although this model is designed to enhance access and facilitate referrals to higher levels of care, gaps in staffing, medical equipment, and transport infrastructure continue to undermine service delivery, especially outside urban centres [9]. Specialised healthcare services remain mainly concentrated in Kampala and a few regional urban hubs, underscoring the need for innovative strategies to address distance and capacity constraints for rural and hard to reach population [5].

Telemedicine, defined as the use of information and communication technologies (ICTs) to deliver healthcare services remotely, has been piloted and adopted in Uganda by both private and public actors [10–12]. The Ugandan Ministry of Health (MoH) has incorporated telemedicine into national policy frameworks, including the National eHealth Policy (2016) and the National eHealth Strategy (2017–2021), which identify telemedicine as a key approach to improving healthcare access and efficiency [13,14]. In line with these policies, the MoH has supported telemedicine initiatives in selected national and regional referral hospitals to facilitate remote consultations, specialist support, and timely patient care, particularly for people with chronic conditions and those in hard-to-reach areas [15]

Private sector innovators have also played a significant role. Rocket Health Uganda has pioneered 24/7 virtual consultations, laboratory sample collection, and medicine delivery services [16]. Public tertiary institutions, including the Uganda Cancer Institute (UCI) and Butabika National Referral Mental Hospital (BNRMH), have used telemedicine to expand specialist reach and strengthen follow-up care [17]. Telemedicine uptake increased markedly during the COVID-19 pandemic, when movement restrictions and physical distancing measures highlighted its importance in maintaining continuity of care, particularly for patients requiring chronic care [16,18].

### Telemedicine potential and constraints

Globally, telemedicine has demonstrated potential to reduce travel and waiting times, improve continuity of care, and facilitate access to specialist input, particularly in settings where local expertise is limited [19]. However, in Uganda, its effectiveness depends on how key stakeholders, patients, providers, and policymakers, perceive, accept, and experience it in routine practice [19]

Factors influencing acceptance include perceived usefulness, ease of use, digital literacy, trust in remote assessment, and confidence in referral pathways for in-person care when necessary [20]. Acceptance of telemedicine’s reliability, clinical quality, privacy, and confidentiality plays a central role in shaping its utilization [21]. Positive perceptions promote uptake and user satisfaction, whereas concerns about misdiagnosis, impersonal communication, and data security may limit engagement [22]. Acceptance is also shaped by socio-cultural norms, prior experiences with the health system, and expectations regarding interpersonal care [21]

Stakeholder experiences of how telemedicine functions in practice provide insight into both enabling factors and barriers. Studies from diverse contexts report benefits such as convenience, cost savings, and improved access, alongside challenges including poor connectivity, limited digital skills, and the inability to conduct physical examinations remotely [23,24]

In Uganda, emerging evidence from initiatives such as Short Message Service (SMS)-based follow-up and Artificial Intelligence (AI)-supported screening indicates high acceptability for specific services but also highlights concerns related to sustainability, workload, and interoperability [24]. Despite increasing interest, there is limited Uganda-specific qualitative research exploring the perspectives of providers, service users, and policymakers on telemedicine acceptance and experiences. Understanding these dimensions is essential to ensure that telemedicine is equitable and effectively integrated into existing care pathways. Therefore, this study explored the acceptance and experiences of telemedicine by policymakers, healthcare providers, and service users involved in telemedicine in Uganda.

### Theoretical orientation

In the Ugandan context, where telemedicine is implemented within infrastructural, regulatory, and socio-cultural constraints, the Technology Acceptance Model (TAM) proposed by Fred Davis, was adopted in this study as a sensitising framework rather than a predictive model. The TAM provides a widely used framework for understanding how individuals come to accept and use emerging technologies [25]. The TAM emphasizes perceived usefulness and perceived ease of use as key determinants of attitudes and willingness to engage with a technology [26,27].

In healthcare settings, technology acceptance is further shaped by trust in providers, confidence in clinical quality, concerns about privacy and confidentiality, and the broader health system context [28,29]. While TAM has been widely applied in studies of digital health adoption, its application within qualitative research, particularly in LMIC contexts, remains relatively limited and contextually underexplored [30]. In the Ugandan context, where telemedicine operates amid infrastructural, regulatory, and socio-cultural constraints, applying TAM as a sensitizing framework can help elucidate how stakeholder experiences influence acceptance in practice. This study addresses this gap by exploring stakeholder perspectives across four institutions offering telemedicine in Uganda, using a theory-informed qualitative approach to generate evidence that can inform service design and contribute to ongoing policy discussions in similar LMIC settings.

In this study, we distinguish between experiences, perceptions and acceptance while recognizing their close interrelationship. Stakeholder experiences refer to participants’ accounts of how telemedicine functions in practice, including factors that enable or constrain its use in real-world settings. These experiences shape stakeholders’ perceptions, particularly perceived usefulness and perceived ease of use, the core constructs of TAM. Acceptance is defined as the expressed willingness to use, continue using, or recommend telemedicine services, influenced by these perceptions and contextual factors. This distinction enables us to examine how lived experiences inform perceptions, and how these perceptions shape acceptance, without framing the study as an evaluation of effectiveness or adoption outcomes.

Because the four participating institutions differ in mandate and scale of telemedicine delivery/use, we classify telemedicine services along two dimensions to aid comparison across sites. The first dimension is the purpose of interaction: patient-to-doctor exchanges for direct patient care, doctor-to-doctor exchanges for specialist consultation or case discussion and doctor-to-nurse or other health-worker exchanges for case escalation or decision support. The second dimension is delivery mode: live call or video (synchronous) consultation; store-and-forward (asynchronous) transmission of clinical information such as images or patient data for later review; remote patient monitoring, involving continuous or periodic transmission of health data for clinical oversight; and mobile health, referring to app- or messaging-based communication and follow-up.

Mapped against this framework, the Ministry of Health’s role is centred on policy leadership and regulatory oversight of telemedicine rather than direct service delivery; Rocket Health Uganda delivers predominantly patient-to-doctor care through live call video and mobile-health channels, alongside store-and-forward laboratory and delivery services; the Uganda Cancer Institute uses telemedicine mainly for doctor-to-doctor specialist consultation and patient follow-up; and Butabika National Referral Mental Hospital uses it primarily for patient-to-doctor mental health support and follow-up calls. This classification is used throughout the Methods and Results to situate site-level findings.

## Methods

### Ethics statement

Ethical approval was obtained from the Research Ethics Committee of Uganda Christian University (Application No: UCUREC-2024–1049). Administrative clearance was secured from participating institutions. All participants provided written informed consent, and data were de-identified and securely stored.

### Study setting

We conducted an exploratory qualitative study to examine stakeholders’ telemedicine acceptance and experiences in Uganda, an area that remains relatively under-researched. The study was carried out in Kampala across four purposively selected institutions involved in telemedicine delivery and oversight: the Ministry of Health (MoH), Rocket Health Uganda, Uganda Cancer Institute, and Butabika National Referral Mental Hospital. These sites represent a national policy leadership, private digital health provision, and public specialized referral services.

### Study population and eligibility criteria

Participants included policy-level actors involved in telemedicine regulation (MoH), healthcare providers directly involved in remote service delivery, and service users who had accessed telemedicine services at participating institutions. Eligible KII participants were policymakers or healthcare providers with at least 2 years direct involvement in delivering, coordinating, or regulating telemedicine services at one of the four sites. Eligible FGD participants were adult service users who had received at least one telemedicine consultation in the preceding year. Individuals with no direct telemedicine involvement were excluded.

### Sampling and recruitment

Purposive sampling guided the recruitment of stakeholders with relevant experience, with support from study focal persons. Between November 2024 and February 2025 data was collected using key informant interviews (KIIs) and focus group discussions (FGDs) using semi-structured guides. The four sites were purposively selected, because together they span the three institutional types of interest to this study: national policy leadership (MoH), private digital health provision (Rocket Health), and public specialised referral care (UCI and BNRMH). The trained research assistant together with study focal persons at each site identified and approached potential KII and FGD participants who met the eligibility criteria above; participation was voluntary and each FGD had between 6–8 service users.

### Data collection

Data was collected by two trained research assistants, supervised by the Principal Investigator and coordinators at each participating site. All KIIs and FGDs were conducted on-site in a private, secure room at each facility to protect participant confidentiality and minimize interruptions.

A total of 12 KIIs were conducted across the four study sites: 3 at Butabika National Referral Mental Hospital, 3 at Rocket Health, 3 at Uganda Cancer Institute (UCI), and 3 at the MoH. The number of KIIs per site was guided by data saturation, with recruitment at each site continuing until no new themes or insights emerged from successive interviews.

Three FGDs were conducted to complement the KIIs: one with service users at Butabika Hospital, one with healthcare providers at UCI, and at Rocket Health, capturing both provider and patient perspectives.

Interview guides; S1 and S2 Texts for FGDs and KIIs respectively were informed by the TAM, focusing on perceived usefulness and perceived ease of use, and explored perceived benefits and challenges, usability, trust in remote care, integration with in-person services, and willingness to use or recommend telemedicine. The guides covered all doctor-involved telemedicine domains: doctor-to-doctor (specialist tele-consultation and referral support), doctor-to-patient (direct clinical tele-consultations), and patient-to-doctor (patient-initiated remote contact). Consistent with the classification above, interview guides probed patient-to-doctor experiences in greatest depth, reflecting this as the dominant mode of telemedicine encountered across study sites.

KIIs lasted 30–45 minutes and FGDs 50–60 minutes. All interviews were conducted in a secure setting, audio-recorded with participant consent, and supplemented with field notes. Recordings were transcribed verbatim and translated into English where necessary. Transcripts were then prepared for coding.

### Data analysis

Coding combined deductive (model-informed) and inductive approaches, with themes refined through interactive team discussions. NVivo 12 software was used to support data management. Triangulation across participant groups and data collection methods enhanced credibility. Coding and theme development were led by IS and SS, with independent coding of a subset of transcripts by a second team member and resolution of discrepancies through discussion; credibility was further supported by peer debriefing among the study team and maintenance of an audit trail of coding decisions. Acceptance was defined as expressed willingness to use telemedicine, continued engagement, trust, and willingness to recommend the service. The study followed six phases of thematic analysis as outlined by Virginia Braun and Victoria Clarke [31]: familiarising with the data, generating initial codes, developing themes, reviewing themes, defining and naming themes, and producing the report.

## Results

### Participants demographics

A total of 12 key informant interviews (KIIs) with healthcare providers and MoH officials, alongside 3 focus group discussions (FGDs) with service users of telemedicine services.

Participants represented a range of professional roles including nurses, clinical officers, tele-nurses, doctors, delivery agents, laboratory technicians, and pharmacists from telemedicine services providers as well as service users. MoH respondents were purposively selected from the Division of Health Information (DHI), which is responsible for implementing and coordinating digital health innovations including telemedicine in Uganda. Their inclusion was intended to provide policy level insights on governance, implementation and scale up of telemedicine services as summarized in Table 1.

**Table 1:** Summary of key informant interviews (KIIs) and focus group discussions (FGDs) by site.

Category	Site	Composition	n	Category/Site Total
KII	Uganda Cancer Institute (UCI)	Pharmacist [1], Nurse [1], Medical Doctor [1]	3	3
KII	Rocket Health	Pharmacist [1], Nurse [1], Medical Doctor [1]	3	3
KII	Butabika National Referral Hospital	Pharmacist [1], Nurse [1], Medical Doctor [1]	3	3
KII	Ministry of Health (MoH)	Commissioner [3]	3	3
**KII Subtotal**				**12**
FGD	Uganda Cancer Institute (UCI)	Mixed healthcare providers	1	1
FGD	Rocket Health	Service users	1	1
FGD	Butabika National Referral Hospital	Service users	1	1
**FGD Subtotal**				**3**
**Grand Total (KIIs + FGDs)**				**15**

Participants contributed varied perspectives on the acceptance and experiences of telemedicine services. The thematic analysis generated three overarching themes reflecting stakeholders’ experiences, perceptions, and recommendations regarding telemedicine in Uganda. These themes are: (1) Perceived Benefits of Telemedicine, (2) Perceived Challenges of Telemedicine, and (3) Recommendations for Improvement. Within each theme, several subthemes emerged providing a nuanced understanding of participants’ views. For example, under ‘convenience and accessibility,’ participants described how telemedicine reduced the burden of travelling long distances and improved access for people with disabilities. A focus group participant from Rocket Health also noted that digital reminders support medication adherence. Under ‘Technological Infrastructure and Connectivity,’ stakeholders identified poor internet coverage and language barriers in rural areas as key barriers to equitable telemedicine use. Similarly, in the ‘Policy and Regulation’ subtheme, participants emphasised the importance of developing a national telemedicine policy and data privacy laws to ensure secure and standardised service delivery.

Consistent with the classification introduced earlier, participant accounts across all four sites centred predominantly on patient-to-doctor interactions; doctor-to-doctor and doctor-to-nurse exchanges were referenced mainly by KII respondents describing internal referral and escalation processes rather than by FGD participants. Table 2 maps the main findings under each theme to the corresponding purpose-of-interaction and delivery-mode categories.

**Table 2:** Mapping of telemedicine classification to key findings.

Theme	Purpose of interaction	Delivery mode	Finding
Convenience and accessibility	Patient-to-doctor	Mobile health / live video	Digital reminder systems supporting medication adherence
Reduced need for physical travel	Patient-to-doctor	Live call or video / mobile health	Remote consultation reducing time and transport costs
Technological infrastructure and connectivity	Patient-to-doctor	Live call or video / mobile health	Poor internet coverage and language barriers in rural areas
Policy and regulation	Doctor-to-doctor / patient-to-doctor	All modes	Need for national telemedicine policy and data privacy laws

## Perceived Benefits, challenges, and recommendations for telemedicine in uganda

### Perceived benefits of telemedicine

Participants described telemedicine as improving access to care, convenience, inclusivity, medication adherence, privacy, flexibility, family support, and cost savings.

#### Convenience and accessibility.

Telemedicine reduces the burden of traveling long distances, especially in rural and semi-urban areas with poor road infrastructure. It enhances inclusivity for people with disabilities and supports medication adherence through digital reminder systems.

“*Many people with disabilities feel relieved from navigating long distances and poorly maintained roads, especially in rural or semi-urban areas. When it comes to medication adherence, telemedicine provides digital platforms and applications with reminder options. Some people I’ve interacted with set digital reminders, which help ensure adherence to medication.*” FGD_RH

Acceptance was reported to be higher among digitally literate populations, particularly in urban districts such as Kampala, Masaka, Wakiso, and Mukono.

“*You’ll understand that in urban areas like Kampala, Masaka, Wakiso, Mukono and other big cities, there has been high acceptance in urban and especially those who are familiar with smartphones, finding tele medicine convenient and empowering.*” FGD_RH

#### Reduced need for physical travel.

Participants emphasized that telemedicine saves time and reduces transport costs by limiting unnecessary facility visits, particularly for follow-ups and minor conditions.

“*For the advantages, what I know is that it’s time saving for the patients… someone remains at home and then calls or sends a message and then the doctors call them back… get the test there, for the medication to be delivered.”* KII_RH

#### Enhanced privacy and autonomy.

Virtual consultations were perceived to increase privacy, dignity, and independence, particularly for people with disabilities and individuals concerned about stigma.

“*Tele-medication platforms give you that independence of interacting with the healthcare provider on a one-on-one, which is increasing privacy for a person with disability.”* FGD_RH

#### Flexible service delivery and family support.

Telemedicine was valued for bringing services and medication directly to patients’ homes, ensuring continuity of care. It also allows family members to provide emotional and practical support during treatment.

“*They can find me at home with all the medication… I can just call and they bring an ambulance or the doctor comes.”* FGD_BTBK

“*The biggest advantage… is having your loved ones by your side… You don’t see your loved ones for over 5 months; you are alone here eating porridge.”* FGD_BTBK

#### Cost-effectiveness.

Telemedicine reduces transport expenses and waiting time at health facilities, allowing patients to manage other responsibilities more efficiently.

“*If I’m able to get that same help online, it really saves me from wasting a lot of time sitting in line… It also saves me transport*.” FGD_RH

### Perceived challenges of telemedicine

#### Technological infrastructure and connectivity.

Limited internet access, poor network coverage, lack of smartphones, and language barriers were identified as major barriers, particularly in rural areas.

“*In village settings… it may come with a lot of problems in terms of communication and networks and the language barrier*.” FGD_UCI

#### Risk of misdiagnosis and communication gaps.

Participants expressed concern that absence of physical examination may compromise diagnostic accuracy and trust, particularly among older and rural populations.

“*Many Ugandans… prefer direct interaction… There is a belief that physical presence enhances trust and diagnosis accuracy.”* KII_MOH

“*We shall be mixing up diagnosis… If a child has flu and cough but is also developing measles, how do you describe the skin*?” KII_UCI

### Recommendations for improving telemedicine

#### Public awareness and multiple helplines.

Participants recommended mass media campaigns and increasing the number of toll-free and paid helplines to reduce congestion.

“*Put some advertisements on TV… put more numbers, not only one toll-free number… make others free, but others also pay.”* FGD_BTBK

#### Expansion beyond urban centers.

Stakeholders called for scaling telemedicine pilots to rural and peri-urban areas to promote equity in access.

*“We have engaged in several pilot projects in urban settings… However, these efforts remain limited outside major urban centers.”* KII_MOH

#### Policy and regulation.

Respondents emphasized the need for a comprehensive national telehealth policy, including clear data protection and privacy regulations.

*“We need a comprehensive health policy… laws that help to guide this… to make sure that patient data is really kept from leaking.”* FGD_RH

#### Clinical protocols and training.

Clear clinical guidelines and structured training programs for healthcare providers were recommended to improve service quality and client management.

“*Establish robust protocols and guidelines… engage trainings for healthcare providers… to reduce the other challenges.”* KII_RH

#### Infrastructure development.

Improving internet coverage and communication networks, especially in villages, was seen as essential for expanding telemedicine reach.

“*Improved coverage… especially in the villages… setting up internet infrastructure… can enable everybody to reach out*.” FGD_RH

## Discussion

This study explored stakeholder acceptance and experiences of telemedicine among policymakers, healthcare providers, and service users in Uganda. Overall, telemedicine was considered acceptable when it improved convenience, reduced travel, enhanced privacy, and supported continuity of care. Participants consistently valued its time-saving nature, cost reductions, and the autonomy it offers in seeking care. For people with disabilities and individuals with mobility challenges, the ability to access services without travelling long distances or navigating poorly maintained roads was described as transformative. Flexible service delivery, including remote consultations, follow-up calls, and home delivery of medication was perceived as empowering and patient centered.

Acceptance and experience also varied by level of care and institutional type. At the Uganda Cancer Institute, a tertiary specialist facility offering doctor-to-doctor consultation alongside patient follow-up, telemedicine was valued mainly for extending specialist reach for patients who would otherwise face long travel for chronic condition management, and concerns about diagnostic accuracy without physical examination were most pronounced among these providers. At Butabika National Referral Mental Hospital, where telemedicine centred on direct patient-to-doctor mental health support and follow-up calls rather than doctor-to-doctor referral, participants valued continuity of psychiatric care and reduced travel burden, while also raising practical concerns such as the cost of toll-free helplines.

At Rocket Health, a private digital service delivery model built around live video and mobile-health patient-to-doctor consultations, participants emphasised speed, convenience, and round-the-clock availability, with fewer concerns about diagnostic limitations given its focus on lower-acuity and follow-up care. Ministry of Health respondents, reflecting their policy leadership role rather than direct service delivery, focused instead on the absence of a comprehensive national regulatory framework as the main constraint on scale-up across all institutional types. These contrasts suggest that acceptance is shaped not only by individual perceptions of usefulness and ease of use, but by the clinical acuity and institutional mandate of the service through which telemedicine is delivered.

Interpreted through the TAM, acceptance of telemedicine was strongest when stakeholder experiences reinforced both perceived usefulness and perceived ease of use. Convenience, reduced travel, privacy, and continuity of care were key drivers of perceived usefulness. Digital literacy, reliable internet connectivity, and clear provider–patient communication enhanced perceived ease of use. Where these enabling conditions were absent, particularly in rural settings, acceptance was more cautious, even when participants acknowledged its potential benefits.

The finding that telemedicine reduces the burden of accessing care during emergencies aligns with evidence from sub-Saharan Africa where telemedicine played a critical role in maintaining service access during the COVID-19 pandemic, particularly in underserved settings [33]. Similarly, Okereafor et al. (2020) highlighted telemedicine’s role in bridging service delivery gaps by minimizing face-to-face consultations during lockdowns [32]. These findings are consistent with stakeholder accounts in Uganda, where telemedicine was associated with enhanced flexibility and continuity of care [33].

Participants also emphasized cost and time savings, a finding consistent with previous studies [20,33]. Similar findings have been reported in Uganda where patients and healthcare workers valued telehealth for reducing transport costs and waiting times. Comparable conclusions have been reported elsewhere in the region suggesting a growing consensus on the convenience and economic value of virtual care models [18].

Despite these benefits, several important barriers were identified. Digital illiteracy and limited patient awareness, particularly in rural areas, constrained equitable access to telemedicine services. These findings mirror research from Kenya which identified limited ICT skills among both patients and providers as a key barrier to digital health adoption [34]. Without targeted digital literacy initiatives, telemedicine may exacerbate existing health inequities.

Infrastructural challenges were also prominent. Poor internet connectivity, unreliable electricity, and weak network coverage were repeatedly cited as barriers to effective telemedicine delivery. Similar challenges have been documented in other LMIC settings [10,12]. These structural limitations remain a major barrier to the scalability and sustainability of telemedicine across East Africa.

Policy and regulatory uncertainty also shaped stakeholder perceptions. Some participants expressed concern about the absence of a comprehensive national regulatory framework for telemedicine. Comparable concerns have been documented where unclear policy environments reduced user confidence [34]. While countries such as South Africa have integrated telemedicine into formal regulatory structures, Uganda’s evolving policy landscape continues to create uncertainty for providers and implementers. Strengthening governance frameworks, including data protection and clinical guidelines, may enhance trust and institutional confidence in telemedicine systems [35].

Importantly, much of the existing literature has focused on telemedicine during public health emergencies. This study contributes to the emerging discourse on long-term integration and sustainability of telemedicine within routine healthcare delivery. Stakeholders accepted telemedicine when it meaningfully reduced the burden of accessing care, however, their experiences also revealed ongoing concerns related to clinical examination, communication clarity, digital literacy, and infrastructure reliability.

Telemedicine acceptance in Uganda appears to be shaped by a dynamic interaction between perceived usefulness, perceived ease of use, and structural enabling factors. Strengthening user support systems, investing in digital infrastructure, enhancing provider training, and establishing clear regulatory frameworks will be critical to sustaining positive user experiences and promoting equitable scale-up of telemedicine services.

## Study strengths and limitations

This study has several limitations. Most participants were drawn from urban-based institutions in Kampala where telemedicine services are relatively established. As a result, the findings may not fully reflect the realities of rural or underserved areas, where digital infrastructure, internet connectivity, and access to smartphones are more limited. Perceptions of usefulness and ease of use in these early-adopter settings may therefore differ substantially from those in resource-constrained environments.

The study focused on sites that were already implementing telemedicine services. Consequently, perspectives from facilities without telemedicine programs, as well as from individuals who have never used such services, were not included. The absence of these perceptives may limit a comprehensive understanding of barriers to initial adoption and reasons for non-use. Together, these limitations may affect the broader generalizability of the findings to the wider Ugandan healthcare context, particularly in rural and low-resource settings.

## Conclusion

Overall, participants described telemedicine as acceptable, particularly when it improved convenience, reduced travel burdens, lowered costs, supported continuity of care, and enhanced privacy and autonomy. These benefits were especially meaningful for persons with disabilities and individuals living far from health facilities. The positive relationship between satisfaction and frequency of use suggests that continued exposure to telemedicine may strengthen confidence and improve user experience over time.

Stakeholders also reported important challenges that shape day-to-day experiences with telemedicine. These included limited internet connectivity, low digital literacy, concerns about diagnostic accuracy, and gaps in policy and regulatory guidance. Such barriers were more pronounced in rural and resource-constrained settings, where digital access remains uneven.

In conclusion, acceptance of telemedicine among policymakers, healthcare providers, and users is closely linked to its perceived usefulness and ease of use within the local context. Strengthening infrastructure, enhancing provider training, improving digital literacy, and establishing clear national policy frameworks will be essential to addressing current challenges. With these enabling conditions in place, telemedicine can move beyond an emergency response strategy and become an integrated and sustainable component of Uganda’s healthcare delivery system.

## Supporting information

S1 Text Focus group discussion (FGD) guide. (DOCX)

S2 Text Key informant interview (KII) guide. (DOCX)

S1 Data Data excel. (XLSX)
