# Supplementary material for: Stakeholder perspectives on telemedicine acceptance and experiences in Uganda: A qualitative study
Source: PLOS Glob Public Health. 2026 Oct 7;6(10):e0007047. doi: 10.1371/journal.pgph.0007047 (PMC13645091; doi:10.1371/journal.pgph.0007047)
Supplement: Supplementary file 1 [file pgph.0007047.s001.docx]

**Focus Group Discussion (FGD) Guide**

*Stakeholder Perspectives on Telemedicine Acceptance and Experiences in Uganda: A Qualitative Study*

Study Aim: To explore stakeholders' acceptance, perceptions, and experiences about telemedicine services in Uganda.

## **Introduction**

Thank you for participating in this discussion. We aim to investigate stakeholders' acceptance, perceptions, and experiences regarding telemedicine services in Uganda. There are no right or wrong answers, we are interested in your honest experiences and opinions. This discussion will take approximately 60-90 minutes.

## **Warm-Up**

1. Before we begin, could each of you briefly share what comes to mind when you hear the word 'telemedicine'?
2. Who here has used, or been involved in delivering, a telemedicine service? Can you briefly describe it?

## **Section A: Acceptance of Telemedicine**

3. What are your thoughts on telemedicine and how it has been taken up here in Uganda?

*Probe: What factors encourage or hinder people from adopting it?*

4. How willing would you be to use, or continue using, telemedicine services, and to recommend them to others?

*Probe: What would make you more or less willing to recommend it?*

5. How do cultural or social attitudes here influence whether telemedicine is accepted or not?

*Probe: What role does trust play in this?*

*Probe: How could telemedicine be better adapted to fit local norms and expectations?*

## **Section B: Perceptions of Telemedicine**

6. How do you perceive the convenience of telemedicine compared to traditional, in-person healthcare?

*Probe: How accessible do you find telemedicine services to be?*

*Probe: What specific conveniences or inconveniences have you noticed?*

7. What is your perception of how effective telemedicine is?

*Probe: Have you noticed improvements in how care is delivered? Are there gaps?*

*Probe: How is trust affected by concerns about privacy and data security? What could improve that trust?*

## **Section C: Experiences with Telemedicine (Objective 3)**

8. Can you describe a specific time you reached out to, or were contacted by, someone remotely as part of getting or giving care -- what happened, from start to finish?

*Probe: What prompted that contact, and how did it unfold?*

*Probe: How easy or difficult was it to get things resolved that way?*

9. Were there moments when an in-person visit or physical examination felt necessary but wasn't possible remotely? What happened in that situation?
10. For those of you working together as a team -- how do you coordinate with one another when care crosses between different people, such as a colleague, a specialist, or someone handling medication?

*Probe: Can you describe an example of that kind of coordination or handover?*

*Probe: What communication challenges come up in those moments?*

11. How would you describe communication during these remote interactions, compared with face-to-face ones?

*Probe: Have there been instances of miscommunication or delay? How did that compare with in-person consultations?*

12. How satisfied have you been, overall, with your experiences with telemedicine?

*Probe: Are there experiences that stand out, positively or negatively?*

*Probe: What improvements would you suggest?*

## **Closing: Recommendations for Improvement**

13. What strategies could make telemedicine more accessible across Uganda?

*Probe: How can infrastructure or policy support be improved?*

*Probe: Are there specific regions or populations that need to be prioritized?*

14. What innovations or changes would you recommend for telemedicine going forward?

*Probe: How can collaboration between different people or institutions involved be strengthened?*

*Probe: What technologies or features would you add?*

15. Is there anything else the group would like to share about telemedicine in Uganda that we haven't covered?

**Thank you for your time and valuable contributions to this discussion.**
