# Supplementary material for: Stakeholder perspectives on telemedicine acceptance and experiences in Uganda: A qualitative study
Source: PLOS Glob Public Health. 2026 Oct 7;6(10):e0007047. doi: 10.1371/journal.pgph.0007047 (PMC13645091; doi:10.1371/journal.pgph.0007047)
Supplement: Supplementary file 2 [file pgph.0007047.s002.docx]

**Key Informant Interview (KII) Guide**

*Stakeholder Perspectives on Telemedicine Acceptance and Experiences in Uganda: A Qualitative Study*

Study Aim: To explore stakeholders' acceptance, perceptions, and experiences about telemedicine services in Uganda.

## **Introduction**

Thank you for participating in this study. We aim to investigate stakeholders' acceptance, perceptions, and experiences regarding telemedicine services in Uganda. Your insights will be invaluable for understanding telemedicine's implementation and utilization. This interview will take approximately 30-45 minutes.

## **Background**

1. Could you describe your current role and how long you have been in this position?
2. In your day-to-day work, how (if at all) do you make use of telemedicine?

*Probe: Who do you typically interact with through telemedicine -- other doctors, nurses, pharmacists, patients, or others?*

## **Section A: Acceptance of Telemedicine (Objective 1)**

3. Can you share your thoughts on telemedicine and how it has been taken up here in Uganda?

*Probe: What factors encourage or hinder its adoption?*

*Probe: Have you personally used or promoted telemedicine services? If yes, how?*

4. How willing are you to make use of, or recommend, telemedicine services to others in your field?

*Probe: What factors influence your willingness to use or recommend it?*

5. How do cultural or social attitudes here in Uganda influence whether telemedicine is accepted or not?

*Probe: What role does trust in technology play?*

*Probe: How can telemedicine be better adapted to local norms and expectations?*

## **Section B: Perceptions of Telemedicine (Objective 2)**

6. How do you perceive the effectiveness and reliability of telemedicine services?

*Probe: What specific aspects do you find beneficial or limiting?*

*Probe: How does telemedicine compare to traditional, in-person ways of working?*

7. Can you describe a time you sought input from, or gave input to, someone else remotely as part of a patient's care -- for example another clinician, a specialist, or a colleague?

*Probe: What platform or method was used?*

*Probe: How did that remote input shape how the case was managed?*

8. How do you perceive the convenience and accessibility of telemedicine compared to in-person services?

*Probe: What specific conveniences or inconveniences have you noticed?*

9. What is your perception of how telemedicine affects trust between you and the people you serve or work with?

*Probe: How is trust affected by concerns about data privacy and security?*

*Probe: What could enhance that trust?*

## **Section C: Experiences with Telemedicine (Objective 3)**

10. How would you describe your day-to-day experience using or supporting telemedicine services?

*Probe: What challenges have you faced in navigating telemedicine platforms or processes?*

*Probe: Are there any features or practices that make things easier?*

11. Can you walk me through what a typical remote interaction looks like for you -- from when it starts to when it's resolved?

*Probe: What happens when something arises that would normally need an in-person visit or a physical examination?*

*Probe: How do you handle handovers or coordination with others involved in that patient's care?*

12. How would you describe communication during these remote interactions, compared with face-to-face ones?

*Probe: Have there been instances of miscommunication or delay? What happened?*

13. How satisfied are you, overall, with how telemedicine has worked in your experience?

*Probe: Are there memorable experiences that stand out, positively or negatively?*

*Probe: What improvements would you suggest?*

## **Closing: Recommendations for Improvement**

14. What strategies could make telemedicine more accessible across Uganda?

*Probe: How can infrastructure or policy support be improved?*

*Probe: Are there specific regions or populations that need to be prioritized?*

15. What innovations or changes would you recommend for telemedicine going forward?

*Probe: How can collaboration between different cadres or institutions be strengthened?*

*Probe: What technologies or features would you add?*

16. Is there anything else you'd like to share about telemedicine in Uganda that we haven't covered?

**Thank you for your time and valuable insights.**
